# Simplified and highly-reliable automated production of [^18^F]FSPG for clinical studies

**DOI:** 10.1186/s41181-023-00200-8

**Published:** 2023-07-24

**Authors:** Mai Lin, Robert T. Ta, H. Charles Manning

**Affiliations:** 1 Cyclotron Radiochemistry Facility, The University of Texas MD Anderson Cancer Center, Houston, TX 77054 USA; 2 Department of Cancer Systems Imaging, The University of Texas MD Anderson Cancer Center, Houston, TX 77030 USA

**Keywords:** [^18^F]FSPG, Automation, Radiopharmaceutical, PET

## Abstract

**Background:**

(S)-4-(3-^18^F-Fluoropropyl)-L-Glutamic Acid ([^18^F]FSPG) is a positron emission tomography (PET) tracer that specifically targets the cystine/glutamate antiporter (xc^−^), which is frequently overexpressed in cancer and several neurological disorders. Pilot studies examining the dosimetry and biodistribution of [^18^F]FSPG in healthy volunteers and tumor detection in patients with non-small cell lung cancer, hepatocellular carcinoma, and brain tumors showed promising results. In particular, low background uptake in the brain, lung, liver, and bowel was observed that further leads to excellent imaging contrasts of [^18^F]FSPG PET. However, reliable production-scale cGMP-compliant automated procedures for [^18^F]FSPG production are still lacking to further increase the utility and clinical adoption of this radiotracer. Herein, we report the optimized automated approaches to produce [^18^F]FSPG through two commercially available radiosynthesizers capable of supporting centralized and large-scale production for clinical use.

**Results:**

Starting with activity levels of 60–85 GBq, the fully-automated process to produce [^18^F]FSPG took less than 45 min with average radiochemical yields of 22.56 ± 0.97% and 30.82 ± 1.60% (non-decay corrected) using TRACERlab™ FXFN and FASTlab™, respectively. The radiochemical purities were > 95% and the formulated [^18^F]FSPG solution was determined to be sterile and colorless with the pH of 6.5–7.5. No radiolysis of the product was observed up to 8 h after final batch formulation.

**Conclusions:**

In summary, cGMP-compliant radiosyntheses and quality control of [^18^F]FSPG have been established on two commercially available synthesizers leveraging high activity concentration and radiochemical purity. While the clinical trials using [^18^F]FSPG PET are currently underway, the automated approaches reported herein will accelerate the clinical adoption of this radiotracer and warrant centralized and large-scale production of [^18^F]FSPG.

## Background

Positron emission tomography (PET) is both a medical and research tool used in pre-clinical and clinical settings. It is widely applied in the imaging of tumors and the search for metastases within the field of clinical oncology, and for the clinical diagnosis of certain neurological disorders. Among all radiopharmaceuticals for PET imaging, the glucose analogue [^18^F]fluorodeoxyglucose ([^18^F]FDG) is the most heavily used to date in oncological applications. However, there are clinical gaps in the effectiveness of [^18^F]FDG PET, and increasingly, PET tracers targeting very specific molecular and even ‘druggable’ pathways are required. As a result, there is a great demand for additional radiopharmaceuticals with unique targeting capability for molecular imaging.

Amino acids play a variety of critical roles in many cellular functions. Due to an accelerated growth rate in cancer cells, the demand for amino acids is elevated as well. Consequently, imaging radiopharmaceuticals based on radiolabeled amino acid analogues have long been appreciated to provide information regarding protein metabolism of malignant cells. Recently, a glutamate-based tracer, (S)-4-(3-[^18^F]fluoropropyl)-L-glutamic acid ([^18^F]FSPG), has started to receive great attention as it has been shown to specifically target the cystine/glutamate antiporter (xc^−^). The xc^−^ antiporter is a plasma membrane transporter mediating cellular uptake of cystine in exchange for intracellular glutamate with an equal stoichiometry ratio (Lo et al. 2008). Although cysteine plays important roles in protein synthesis and in maintaining redox balance, de novo biosynthesis or a catabolic supply of cysteine is not sufficient to meet the high demand for antioxidant synthesis under conditions of oxidative stress such as in cancer and neurological disorders, which drives xc^−^ activity to ensure cysteine supply (Lewerenz et al. 2013; Koppula et al. 2018; Combs and DeNicola 2019). More recently, Lei et al. (2020) discovered that xc^−^ antiporter activity promotes radioresistance largely through inhibiting ferroptosis, a form of regulated cell death induced by lipid peroxidation . These authors found that the xc^−^ antiporter is a target of BRCA1-associated protein 1 (BAP1) and that BAP1 promotes ferroptosis through repressing xc^−^ antiporter expression, resulting in tumor suppression Zhang et al. (2018). Indeed, BAP1 deletions and loss of function mutations are common in many cancers (Murali et al. 2013; Carbone et al. 2020; Han et al. 2021; Yan et al. 2022), and in these tumors, xc^−^ activity would be likely to provide extracellular cystine and tumor protection. In this way, [^18^F]FSPG PET could provide an indirect measure of BAP1 activity, which could result in a better understanding of therapeutic efficacy during the course of various treatments. Recent clinical studies examining dosimetry and biodistribution of [^18^F]FSPG in healthy volunteers (Smolarz et al. 2013; Mosci et al. 2016) and tumor detection in patients with non-small cell lung cancer (NSCLC), hepatocellular carcinoma, and brain tumors have shown promising results (Baek et al. 2012; Kavanaugh et al. 2016; Mittra et al. 2016; Park et al. 2017; Magarik et al. 2018; Paez et al. 2022; Wardak et al. 2022). Of note, the low uptake in the brain, lung, liver, and bowel renders [^18^F]FSPG an excellent imaging agent characterized by high tumor-to-background ratios (Park et al. 2020).

Producing radiotracers with highly consistent radiochemical yield and quality is required to bring promising radiotracers from bench to bedside. Although several groups have described automated procedures to produce [^18^F]FSPG, previous reports were focused on small-scale production (up to 4.8 GBq of the final product) and with complex modifications of commercially available cartridges (Koglin et al. 2011; Kavanaugh et al. 2016; Mittra et al. 2016; McCormick et al. 2019; Shih et al. 2020; Edwards et al. 2021; Brown et al. 2023), making it challenging to directly implement at a manufacturing facility. Here, we report a detailed and highly robust technical protocol to produce the large-scale, cGMP-compliant radiosynthesis of [^18^F]FSPG using GE TRACERlab™ FXFN and FASTlab™, the most widely used synthesis modules for routine clinical radiopharmaceutical production. Whereas TRACERlab™ FXFN includes 9 reagent reservoirs and a HPLC system for users to modify the module and develop new synthesis strategies, FASTlab™ is a cassette-based module that can better comply to cGMP process. Another major factor that differentiates the FASTlab module from their predecessor is the elimination of the pre-synthesis cleaning requirement, thus a natural reduction in preparation works and time. The protocol detailed herein highlights numerous improvements over prior reports, addressing critical limitations, and can be easily followed by anyone skilled in the art and equipped with common resources.

## Materials and methods

### General

**Fig. 1:**
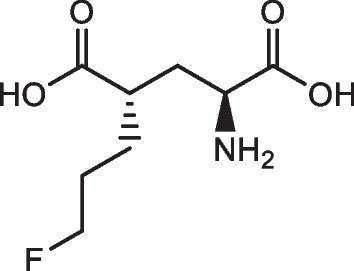
Chemical structure of (4S)-4-(3-fluoropropyl)L-glutamate as the reference standard of [^18^F]FSPG

**Fig. 2:**
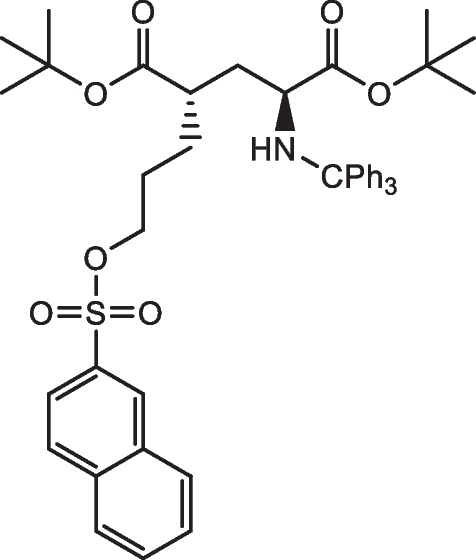
Chemical structure of the precursor to produce [^18^F]FSPG

The automated radiosynthesis of [^18^F]FSPG using GE TRACERlab™ FXFN and FASTlab™ was performed inside the aseptically cleaned COMECER hot cell under cGMP condition. The production of [^18^F]FSPG on GE TRACERlab™ FXFN and FASTlab™ shares common consumables and reagents, in which the reference standard (*(4S)-4-(3-fluoropropyl)L-glutamate*, Prod. # 3194, Fig. 1) and cGMP-grade precursor (*di-tert-butyl (2S,4S)-2-(3-((naphthalen-2 ylsulfonyl)oxy)propyl)-4 (tritylamino) pentanedioate*, Prod. # 3193**, **Fig. 2**)** were purchased from ABX GmbH (Radeberg, Germany), as were the Cryptand-222 (Kryptofix^®^ [2.2.2]) (Prod. # 800) and the preconditioned QMA light cartridges (Prod. # K-920). The Oasis MCX Plus (60 µm LP, Part # 186,003,516) and Alumina N Plus long cartridges (Part # WAT020510) were acquired through Waters (Milford, MA). Supelclean ENVI-Carb SPE graphitized carbon (Cat# 57,094) and 6 mL SPE tubes were obtained from Millipore Sigma (Burlington, MA). Acrodisc^®^ glass membrane filter and sterilized water for injection (SWI) were products of PALL Corp. and Hospira, respectively. All other references of ‘water’ refers to Milli-Q water (18 MΩ cm) taken from a Millipore Milli-Q Integral 5 water purification system and were primarily used in quality control processes. Anhydrous acetonitrile (99.8%) used in the evaporation and labeling steps was from Sigma-Aldrich. Other reagents used in the production process of [^18^F]FSPG include sodium hydroxide (4M NaOH, RICCA), sulfuric acid (1M H_2_SO_4_, Fisher), ethanol (Pharmco), sodium phosphate dibasic dihydrate (Acros Organics); in addition, potassium carbonate and sodium chloride were purchased from Thermo Fisher. The OPA (o-phthalaldehyde) reagent, used in complexation of [^18^F]FSPG and FSPG reference standard for HPLC analysis, was acquired through Agilent.

The Kryptofix stock solution was prepared, in-house, by adding 94.0 ± 1.0 mg of Kryptofix (Cryptand-222) and 9.40 ± 0.10 mg of potassium carbonate in a glass container. The solid mixture was then dissolved with 5 mL of SWI and 5 mL of acetonitrile. The phosphate buffered solution used for final elution of [^18^F]FSPG contained 603 ± 10 mg of sodium chloride and 700 ± 10 mg of sodium phosphate dibasic dihydrate that were dissolved by 100 mL of SWI. All chemicals were used without further purification. Nitrogen and argon gas used primarily in drying and transferring of solutions were provided through Matheson Tri-gas. The automation synthesis on the TRACERlab™ FXFN and FASTlab™ modules were controlled by the TRACERLab FX software and the FASTlab Developer software, respectively.

### Automated synthesis of [^18^F]FSPG

#### Procedure overview

**Fig. 3:**
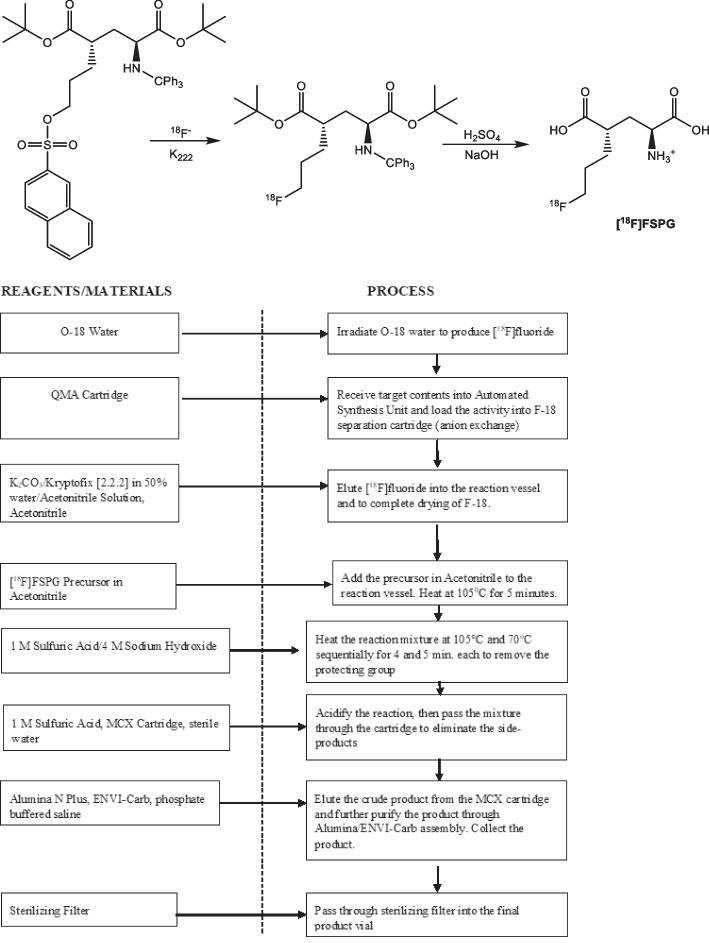
Radiosynthesis scheme and detailed flow chart of producing [^18^F]FSPG

Figure 3 illustrates the radiosynthesis scheme of preparing [^18^F]FSPG. [^18^F]Fluoride was produced by irradiating 2.5 mL of enriched [^18^O]H_2_O with 60 μAh beam current from the 16.5 MeV GE PETrace cyclotron. The [^18^F]Fluoride was separated from the [^18^O]H_2_O by trapping the [^18^F]Fluoride on the preconditioned QMA light cartridge. Following the trapping step, a solution of the Kryptofix stock solution was used to elute the [^18^F]Fluoride from the cartridge into the reaction vessel. The solution was heated to 120 °C to remove the water and acetonitrile initially. Next, the precursor for the reaction, (2*S*,4*S*)di-*tert*-butyl 2-(3((naphthalene-2-ylsulfonyl)oxy)propyl)-4-(tritylamino)pentanedioate dissolved in anhydrous acetonitrile, was added to the reaction vessel. The reaction was heated at 105 °C for 5 min in a closed reaction vessel to promote the substitution of the ^18^F for the sulfonate leaving group. Next, the 1M sulfuric acid solution was added and the reaction was heated at 105 °C for 4 min to reveal the carboxylic acid groups. Hydrolysis occurred as 4.0M sodium hydroxide was added and the reaction was heated to 70 °C for a period of 5 min. The solution was then allowed to cool and a second batch of the 1M sulfuric acid was added to acidify the mixture. The reaction mixture was transferred onto the MCX cartridges connected in series. The cartridges were then washed with SWI to remove impurities. The [^18^F]FSPG was then desorbed from the MCX cartridges with the phosphate buffered solution. The [^18^F]FSPG was further purified by passing the eluent through an Alumina N Sep-Pak cartridge and a SPE column packed with 1.5 g of ENVI-Carb graphite carbon to eliminate remaining [^18^F]fluoride and hydrophilic impurities. The desired product was finally passed through a 0.22 µm vented sterilizing filter into a sterile vial for QC sampling and dose dispensing.

#### GE TRACERlab™ FXFN setup

**Fig. 4:**
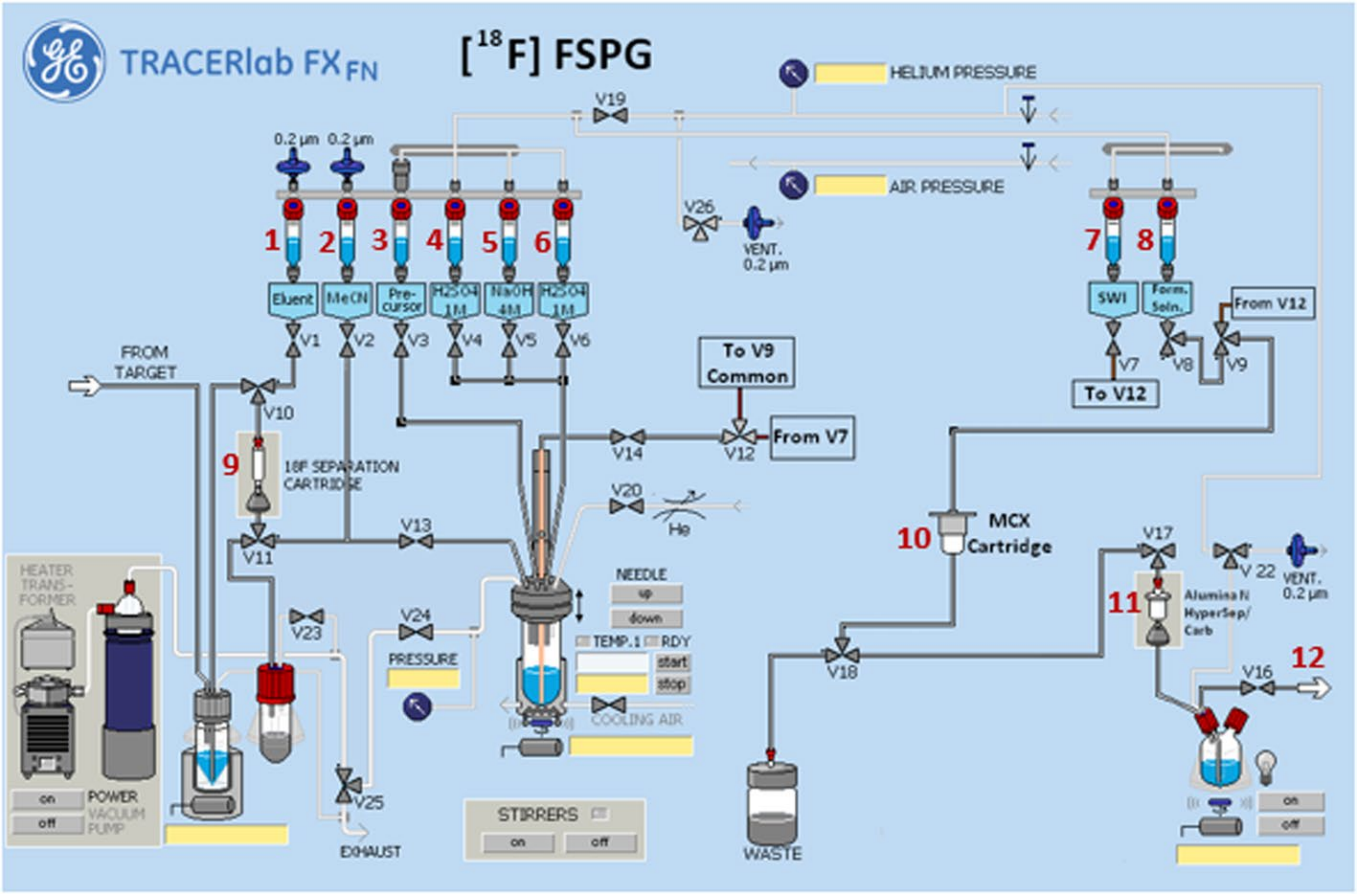
Schematic overview of the [^18^F]FSPG radiosynthesis on the GE TRACERlab™ FXFN module. The numbers denoted in RED, represented the designation of item and position of reagents and consumables indicated on Table 1

**Fig. 5:**
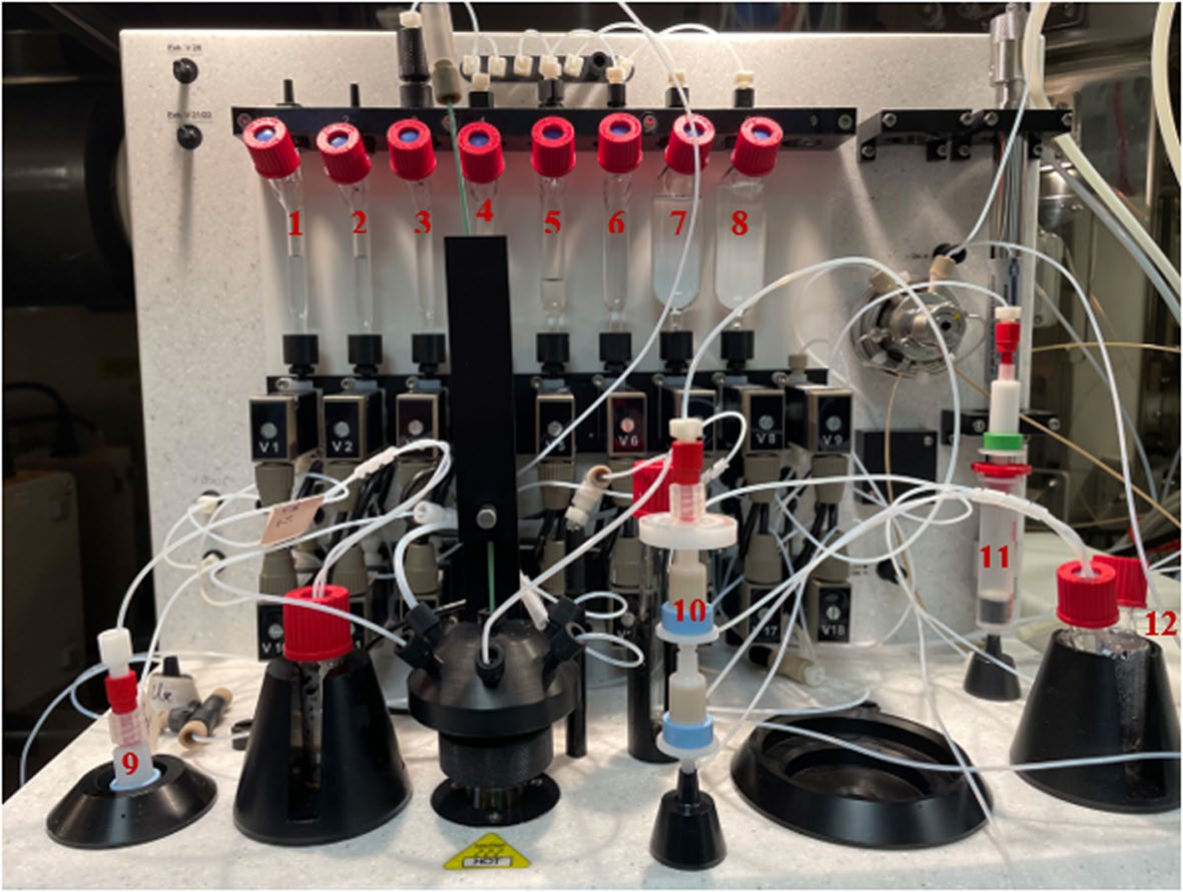
GE TRACERlab™ FXFN module set-up of reagents and cartridge/filter assembly for the radiosynthesis of [^18^F]FSPG

**Fig. 6:**
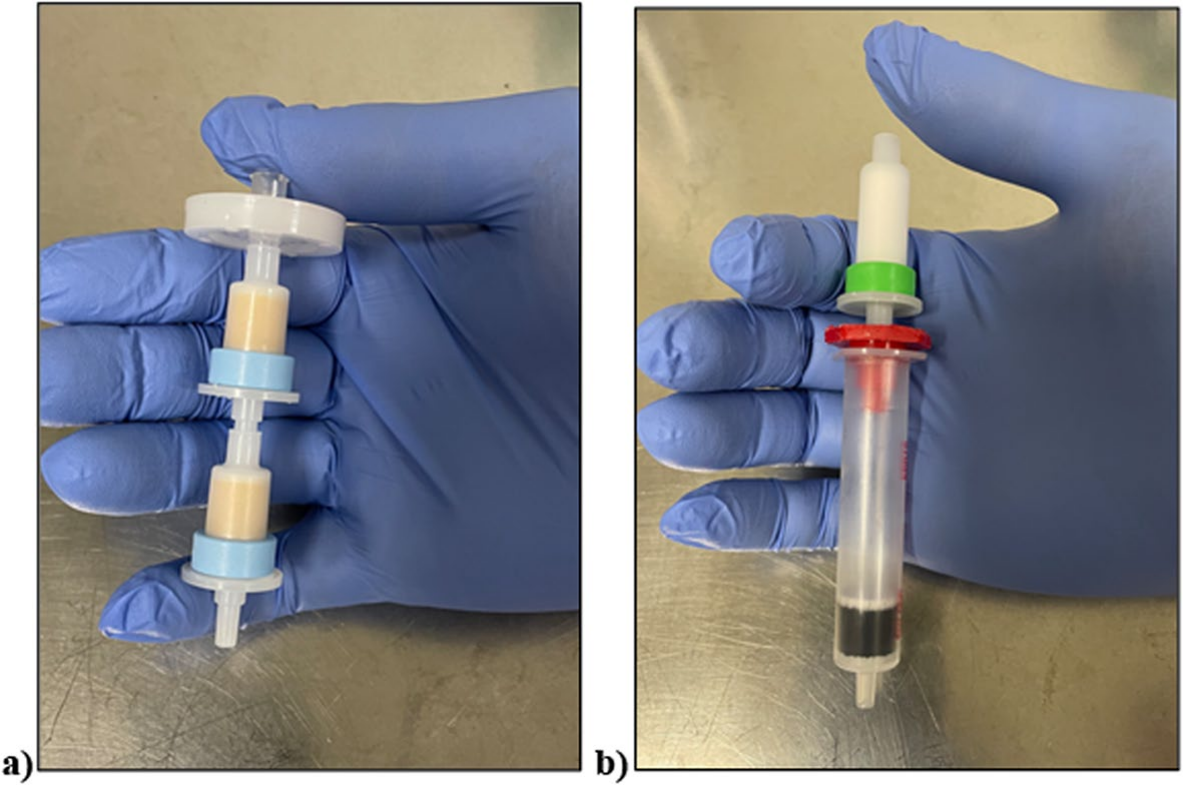
Assembling the Oasis^®^ MCX cartridge/Glass membrane filter set (**a**) and the Alumina N Cartridge/Superclean™ ENVI-Carb™ assembly (**b**). The assemblies were installed and activated only on the day of the synthesis

Figures 4 and 5 illustrates the preparation setup for the radiosynthesis of [^18^F]FSPG for injection. Vials 1 through 8 were loaded with the respective reagents shown on Table 1. At position 9, a pre-conditioned QMA cartridge was installed and used as purchased. Two Oasis^®^ MCX cartridges were attached and conditioned with 10 mL of the formulated phosphate buffered saline, followed by 10 mL of air, then assembled to a single glass membrane filter, as demonstrated in Fig. 6a, and connected to the lines at position 10. To prepare the Alumina N Cartridge/Superclean™ ENVI-Carb™ assembly for the radiosynthesis, approximately 1.5 g of ENVI-Carb resin was weighed and added to a 6 mL Supelco tube and a frit was packed in to secure the resin. The column was then sequentially conditioned with 10 mL of ethanol, followed by 10 mL of the formulated phosphate buffered saline, then dried with 10 mL of air. An Alumina N Plus Long SepPak cartridge was activated with 10 mL of SWI followed by 10 mL of air. The Alumina cartridge was then attached to the top of the ENVI-Carb column, as illustrated by Fig. 6b, and then assembled to the position 11 on the TRACERlab™ FXFN module. A final product vial was connected to the outgoing transfer line of the double-neck vial, as shown in position 12.

**Table 1:** Material and reagent list used in the radiosynthesis of [^18^F]FSPG via TRACERlab™ FXFN module

^*a*^Item #	Reagents or consumables
1	Potassium carbonate/kryptofix QMA elution solution, 0.6 mL
2	Anhydrous acetonitrile, 0.6 mL
3	FSPG precursor, 6 mg dissolved in 1.0 mL anhydrous acetonitrile
4	1M H_2_SO_4_, 2.0 mL
5	4M NaOH, 1.5 mL
6	1M H_2_SO_4_, 4.0 mL
7	Sterile water for injection (SWI), 15.0 mL
8	^*b*^Phosphate buffer solution, 20.0 mL
9	Pre-conditioned QMA light SepPak cartridge, 1 cartridge
10	^*c*^Oasis^®^ MCX cartridge/glass membrane filter assembly, 1 set
11	^*d*^Alumina N plus long cartridge/Superclean™ ENVI-Carb™ assembly, 1 set
12	Final Product vial, 1 vial

^a^Items designation and position are indicated in Fig. 3

^b^Formulation of phosphate buffer solution is specified in the Materials and methods “General” section

^c^Illustration of the Oasis^®^ MCX cartridge/glass membrane filter assembly is shown in Fig. 5a

^d^Illustration of the Alumina N Cartridge/Superclean™ ENVI-Carb™ assembly is shown in Fig. 5b

### GE FASTlab™ setup

**Fig. 7:**
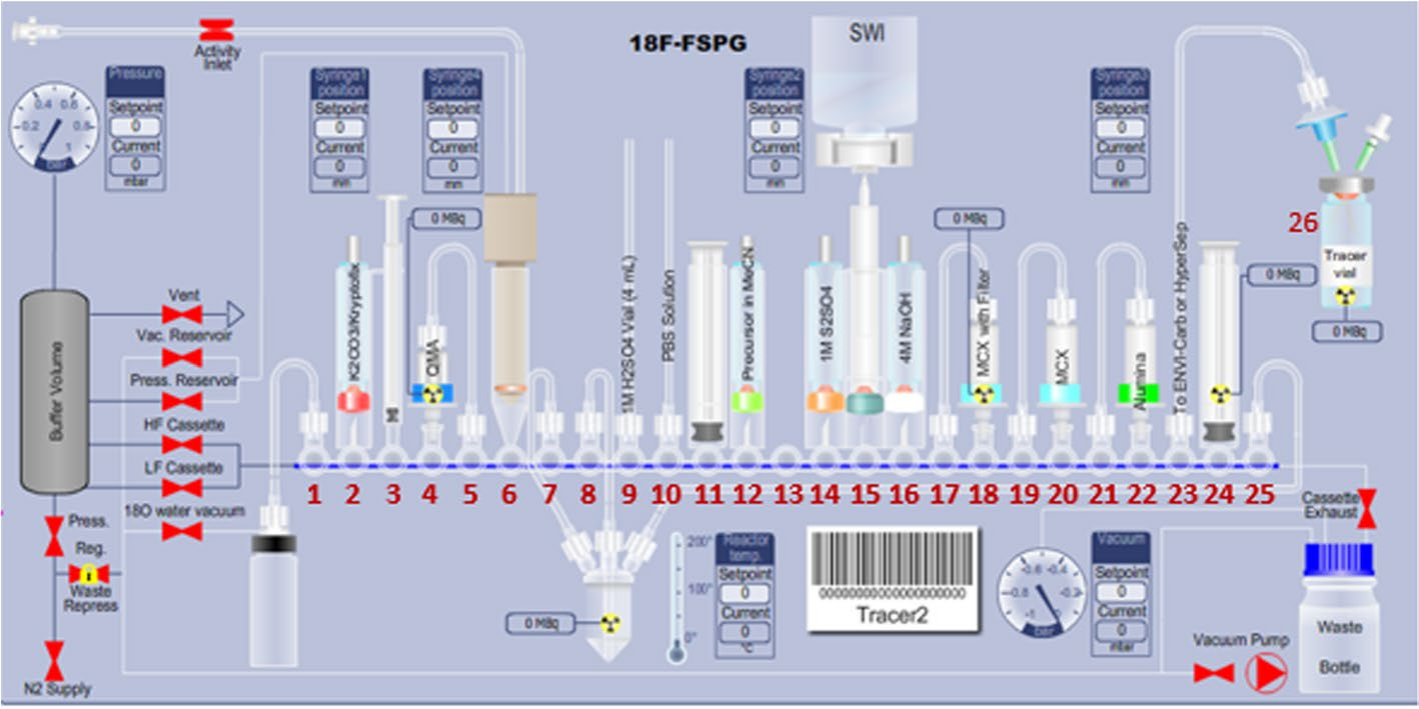
Schematic FASTlab cassette layouts for the radiosynthesis of [^18^F]FSPG. The numbers denoted in RED represent the designation of item and position of reagents and consumables indicated on Table 2

**Fig. 8:**
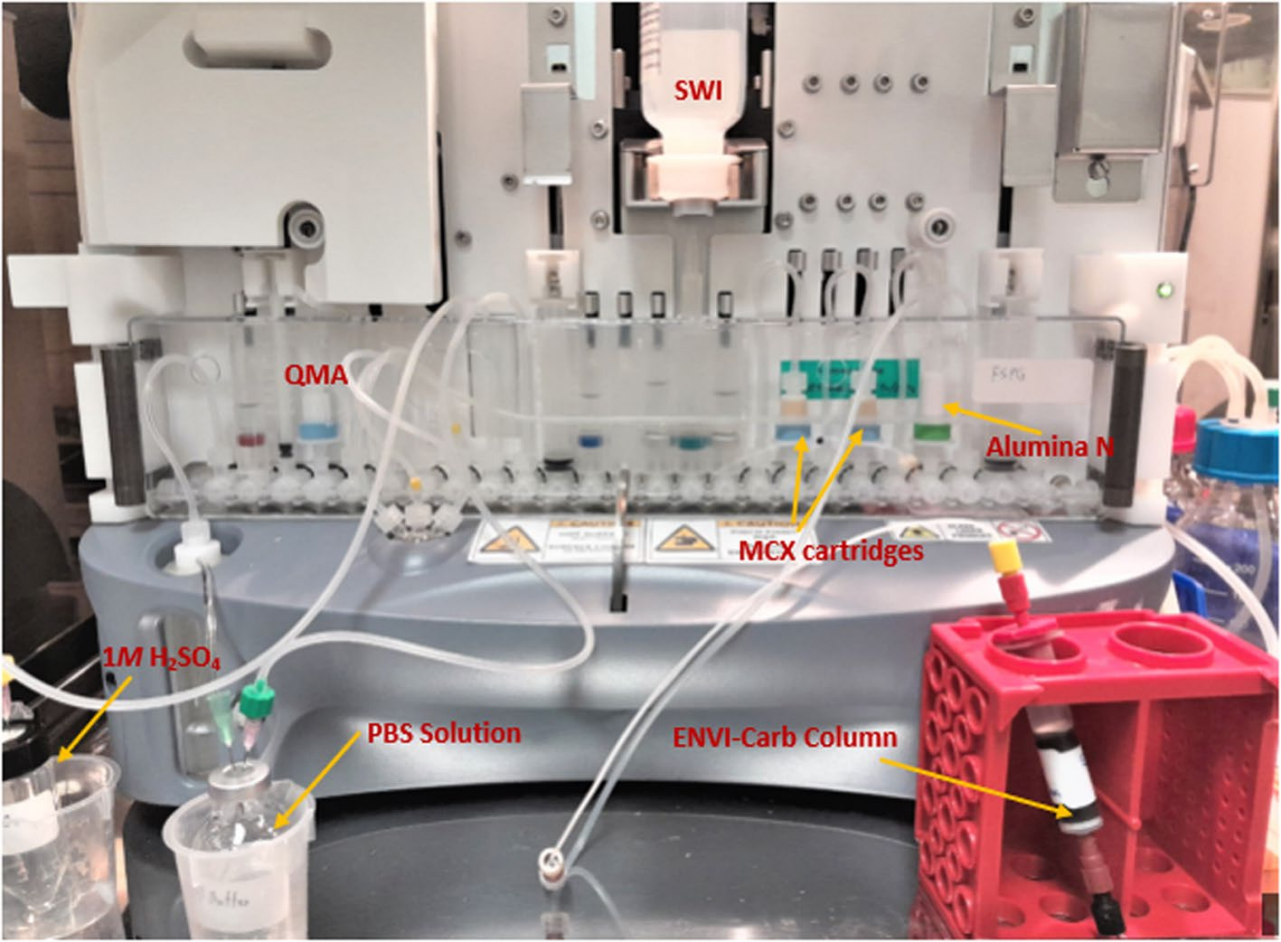
GE FASTlab™ set-up of reagents and cartridges for the radiosynthesis of [^18^F]FSPG

Figures 7 and 8 illustrates the preparation for the radiosynthesis of [^18^F]FSPG for injection using the cassette setup on the *FASTlab™* module. As indicated on Table 2, reagent vials were prepared in 11 mm and 13 mm vials, crimped sealed and loaded to the cassette. The pre-conditioned QMA cartridge was installed and used as purchased. Similarly to the TRACERlab setup, the MCX and Alumina cartridges were conditioned with the same solution sequence and amount. Unlike the setup on the TRACERlab, the two Oasis^®^ MCX cartridges were placed in series in the cassette instead of stacking on top of each other. The FASTlab setup did not require the use of a glass membrane filter and the Alumina N Cartridge was integrated into the cassette as well. In this case, the Superclean™ ENVI-Carb™ column is the only purification column/cartridge that was connected externally. For the radiosynthesis, approximately 1.5 g of ENVI-Carb resin was weighed and added to a 6 mL Supelco tube and a frit was packed in to secure the resin. The column was then sequentially conditioned with 10 mL of ethanol, followed by 10 mL of the formulated phosphate buffered saline, then dried with 10 mL of air. The inlet of the column was connected to the tubing attached to the cassette position CP23. The outlet of the column was connected with the transfer line that would eventually delivered the purified [^18^F]FSPG for injection to the final product vial.

**Table 2:** Cassette position and materials/reagents list used in the radiosynthesis of [^18^F]FSPG via FASTlab™ module

^*a*^Cassette position (CP)	Reagents or consumables
1	Short tubing to ^18^O water collection vial
2	Potassium carbonate/kryptofix QMA elution solution, (11 mm Vial, 0.8 mL)
3	Left hand syringe (LHS), syringe 1
4	QMA light SepPak cartridge, 1 pre-condition cartridge
5	Short tubing to QMA light SepPak cartridge at CP4
6	^18^F Inlet
7	Short tubing to reactor side port
8	Short tubing to reactor center port
9	1M H_2_SO_4_, long tubing connected to external 4.0 mL 1M H_2_SO_4_ vial
10	^*b*^Phosphate buffered saline (PBS), long tubing connected to external 20 mL PBS vial
11	Middle syringe (MS), syringe 2
12	FSPG precursor, (11 mm Vial, 6 -12 mg dissolved in 1.3 mL anhydrous acetonitrile)
13	Unused
14	1M H_2_SO_4_, (13 mm Vial, 2.2 mL)
15	Water Spike/water bag, 100 mL SWI bag
16	4M NaOH (13 mm Vial, 1.7 mL)
17	Short tubing to Oasis MCX cartridge at CP18
18	Oasis^®^ MCX cartridge, 1 cartridge
19	Short tubing to Oasis MCX cartridge at CP20
20	Oasis^®^ MCX cartridge, 1 cartridge
21	Short tubing to Alumina N plus long SepPak cartridge at CP22
22	Alumina N plus long SepPak cartridge, 1 cartridge
23	Connecting tube to Superclean™ ENVI-Carb™ packed column, 1 set
24	Right hand syringe (RHS), syringe 3
25	Long tubing to reactor side port
26	Final Product Vial, 1 vial

^a^Items designation and position are indicated in Fig. 6

^b^Formulation of phosphate buffer solution is specified in the Materials and methods “General” section

#### Quality control method

The characterization of [^18^F]FSPG for injection was performed on the Agilent 1260 HPLC system equipped with a variable wavelength detector and Lablogic NaI radio-detector. The analytical method for determining the identity, chemical and radiochemical purity of the [^18^F]FSPG include the following conditions: The analytical peak separation flow rate was set to 1.5 mL/min through a Phenomenex Luna C-18(2) column (250 mm × 4.6 mm × 5 μm) and the UV detector @ 340 nm. Mobile Phase (A) 40 μM Disodium Phosphate in Water and Mobile Phase (B) Acetonitrile/Methanol/Water (45:45:10) (v/v), with gradient 0–4% B (2 min); 4–12% B (3 min); hold at 12% B (5 min); 12–60% B (5 min); 60–100% B (2 min); hold at 100% B (2 min); 100–0% B (1 min); hold at 0% B (5 min). In this condition, [^18^F]FSPG was eluted at around 13 min.

**Fig. 9:**
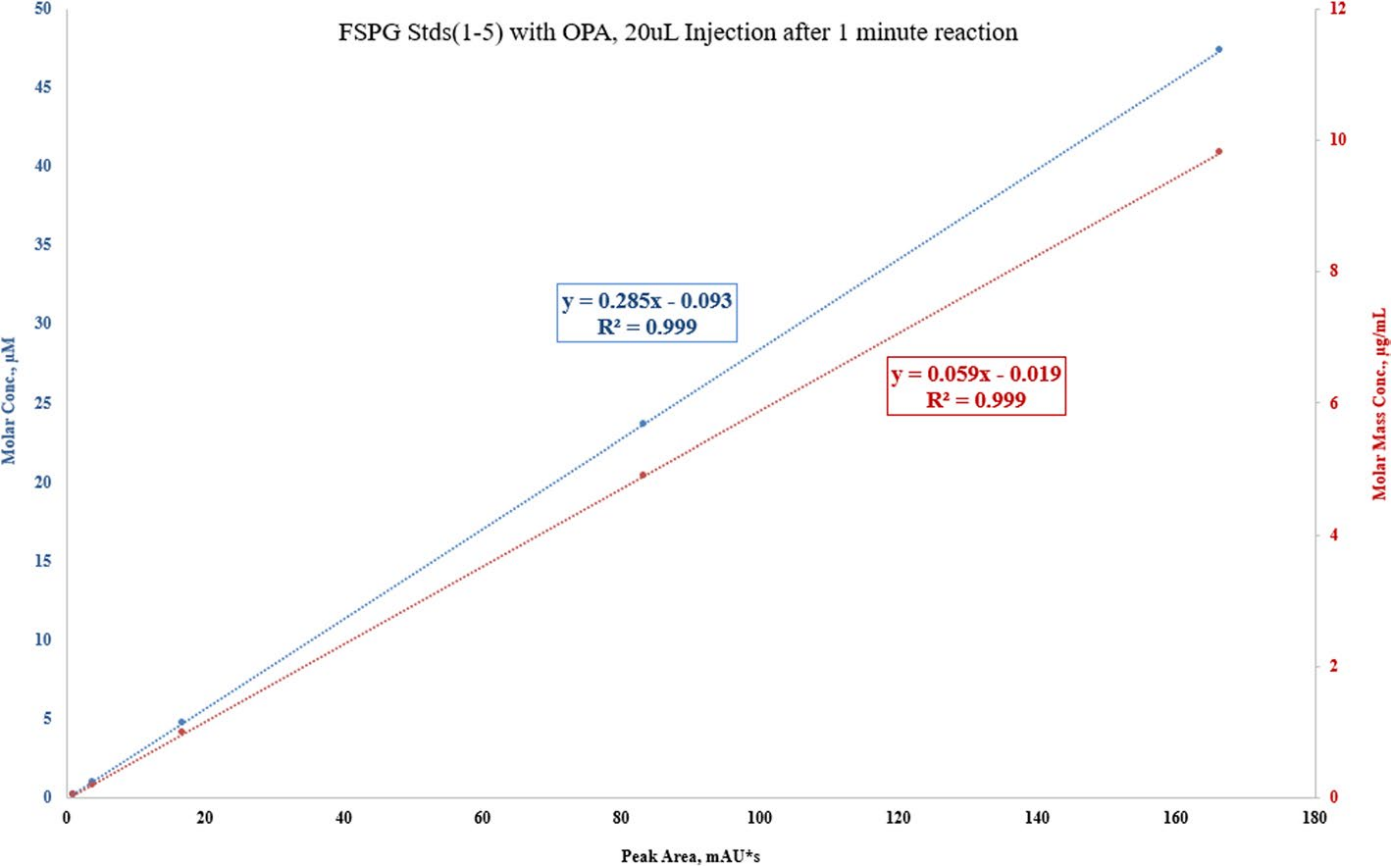
Five (5) point calibration of FSPG reference standard complexation with OPA reagent by HPLC analysis. The blue regression curve represents the molar concentration, whereas the red curve represents the mass concentration, measured against the peak area

**Fig. 10:**
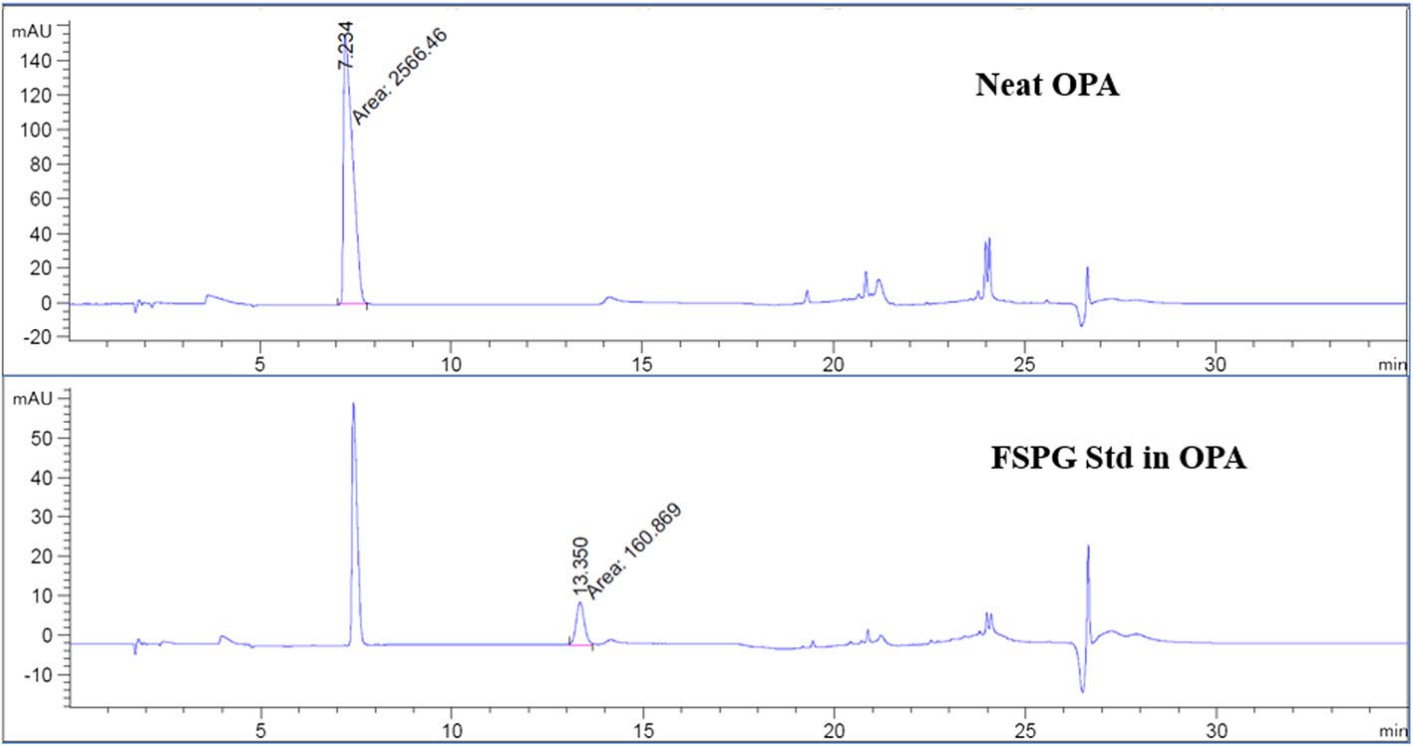
Illustration of UV chromatograms of FSPG reference standard after reaction with OPA reagent by HPLC analysis. Per the method described previously (in “Quality control method” section), the OPA peak elutes around 7 min and FSPG standard elutes at the 13 min region

**Fig. 11:**
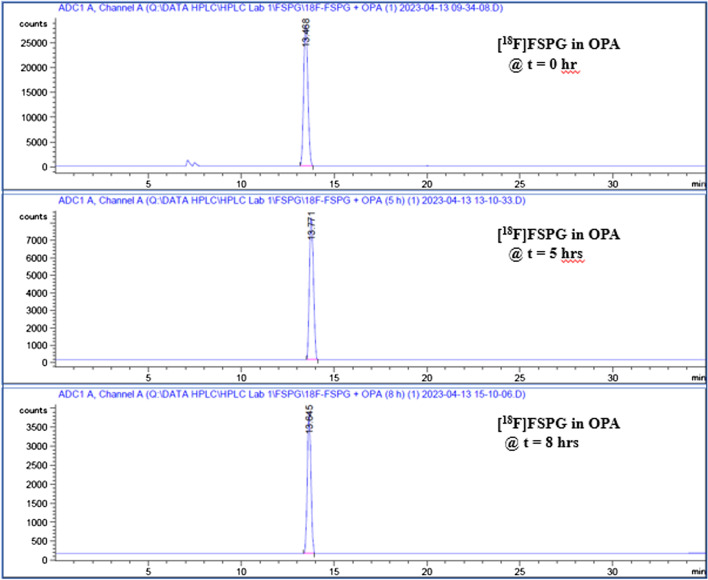
Radio-HPLC chromatograms of [^18^F]FSPG after reaction with OPA reagent. Per the method described previously (in “Quality control method” section), [^18^F]FSPG peak elutes at the 13 min region. The indicated chromatograms verified that [^18^F]FSPG remained stable for 8 h after final batch formulation

The FSPG reference standard was prepared by dissolving 1 mg of cold FSPG standard with 100 mL of phosphate buffered saline. To prepare sample for analysis, 80 µL of FSPG reference standard was first complexed with 20 µL of OPA (o-phthalaldehyde) reagent that has been widely used to react with primary amines of amino acids, peptide, and proteins to enable fluorescent detection and quantitation. The mixture of the reference standard and OPA reagent was then vortexed and allowed to react for 1 min prior to be injected to the HPLC. Similarly, complexation of [^18^F]FSPG with the OPA reagent, in 4:1 ratio, was injected and compared to the previously injected reference standard-OPA complex. Figures 9, 10 and 11 illustrated the data results by HPLC analysis for calibration of FSPG reference standard, identification of OPA and cold FSPG standard, and identity and stability of [^18^F]FSPG for injection, respectively.

The residual solvents in [^18^F]FSPG for injection was determined by Agilent GC system with FID, equipped with GC Column DB200 (30 m × 0.250 mm, 0.50 µm stationary phase thickness). The radionuclidic identity and purity, residual Kryptofix^®^ [_2.2.2_], bacterial endotoxin, sterility. appearance, pH, and filter tests were performed under the USP < 823 > guidelines.

## Results

### ***Automated synthesis of [***^***18***^***F]FSPG***

The production of [^18^F]FSPG was completed within 45 min in the TRACERlab™ FXFN including the trapping of [^18^F]fluoride from the cyclotron to the QMA cartridge on the synthesizer. The results of [^18^F]FSPG synthesis using the TRACERlab are shown in Table 3. The average non-decay-corrected radiochemical yield was 22.56 ± 0.97% with radiochemical purity of 96.31 ± 0.62% when less than 37 GBq of the starting activity and 6 mg of the precursor were applied. However, dramatical reduction in radiochemical yield and slightly lower radiochemical purity were observed when larger starting activity was used. This limit could further be resolved by adding additional amounts of the precursor and ENVI-Carb graphitized carbon for the reaction and final purification process. Because our ultimate goal was to facilitate the centralized production of [^18^F]FSPG for clinical use, we translated these findings to FASTlab™, a cassette-based module, for easier adoption by other radiopharmaceutical manufacturing facilities.

**Table 3:** Radiosynthesis of [^18^F]FSPG via TRACERlab™ FXFN module

Entry	Starting activity (GBq)	Precursor amount (mg)	Total synthetic time (min)	Activity of the obtained [^18^F]FSPG (GBq)	Amount of ENVI-Carb graphitized carbon (g)	Radiochemical purity (%)	Non-decay-corrected radiochemical yield (%)
1	14.80	6	45	3.19	0.5	96.73	21.60
2	24.05	6	45	5.77	0.5	96.79	23.99
3	26.64	6	45	6.07	0.5	95.81	22.79
4	33.56	6	45	7.62	0.5	95.48	22.71
5	37.00	6	45	8.03	0.5	96.72	21.70
6	92.50	6	45	7.51	0.5	93.29	8.12
7	64.01	12	45	12.65	1.5	98.60	19.76
8	64.75	12	45	12.77	1.5	95.99	19.72
9	85.10	12	45	15.80	1.5	95.03	18.57

Similar to automated synthesis performed in the TRACERlab, the production of [^18^F]FSPG was completed within 42 min in the FASTlab. The results of [^18^F]FSPG automated synthesis using a single-use cassette with 12 mg of the precursor and 1.5 g of ENVI-Carb graphitized carbon are shown in Table 4. While a consistently high radiochemical purity (95.96 ± 0.70%) was maintained, the average radiochemical yields was also observed to be significantly higher than from the TRACERlab counterpart, 30.82 ± 1.60% versus 22.56 ± 0.97%, *p* < 0.0001. The much improved radiochemical yields from the FASTlab, in addition to changes previously mentioned, can also be attributed to the compact design of the reactor and more efficient built-in vacuum system in the module compared to the counterpart TRACERlab.

**Table 4:** Radiosynthesis of [^18^F]FSPG via FASTlab™ module

Entry	Starting activity (GBq)	Precursor amount (mg)	Total synthetic time (min)	Activity of the obtained [^18^F]FSPG (GBq)	Amount of ENVI-Carb graphitized carbon (g)	Radiochemical purity (%)	Non-decay-corrected radiochemical yield (%)
1	63.68	12	42	19.31	1.5	95.50	30.32
2	68.97	12	42	22.46	1.5	95.87	32.65
3	59.68	12	42	17.24	1.5	96.98	28.89
4	62.42	12	42	19.61	1.5	95.50	31.42

### Quality control of [^18^F]FSPG for injection

Radio-HPLC analysis of the produced [^18^F]FSPG for injection showed > 95% radiochemical purity. In line with previous reporting, the major impurity can be attributed to [^18^F]Fluoride (Brown et al. 2023). Further analysis after 8 h confirmed that the product remained stable (Fig. 11). The molar activity calculated from the activity yields at EOS for [^18^F]FSPG was in the range of 100–292.3 GBq/μmol. The residual ethanol and acetonitrile concentrations were less than 0.2% and 0.005% (v/v), respectively. The pH was 6.5–7.5 and the bubble point was 70 to 75 psi. In addition, the sterility, pyrogenicity, and all other quality control tests passed specifications based on the USP < 823 > guidelines.

## Discussion

In this study, our work was undertaken to support a variety of clinical trials leveraging [^18^F]FSPG PET. We have achieved the cGMP-compliant automated production of [^18^F]FSPG with optimal synthesis procedure for both GE TRACERlab™ FXFN and FASTlab™ synthesizer. While several commercially available modules for [^18^F]FSPG production have been previously published (Koglin et al. 2011; Kavanaugh et al. 2016; Mittra et al. 2016; McCormick et al. 2019; Shih et al. 2020; Edwards et al. 2021; Brown et al. 2023), our method has consistently produced [^18^F]FSPG at high radiochemical purity and high molar activity. In particular, our adaptation of the SPE purification method was elegantly simplified in setup and with minimal processing time, making it attractive for centralized and large-scale production of [^18^F]FSPG at a manufacturing facility. During our production process, the sulfuric acid and sodium hydroxide were added to the crude reaction mixture to remove trityl and t-butyl protecting groups following radio-fluorination. The solution was then acidified by adding the sulfuric acid a second time prior to passing through the MCX cartridges. [^18^F]FSPG and the non-reacted precursor were trapped on the cartridges whereas most [^18^F]fluoride and water-soluble impurities were eliminated by the MCX, a cation exchange cartridge. Once the phosphate buffered solution was passed through the cartridges, the ion-exchange retention mechanism was terminated because the trapped compounds become negatively charged or unionized. However, due to the reverse-phase characteristic of the MCX cartridge sorbent, most of the non-reacted precursor was retained as a result of its lipophilic nature while [^18^F]FSPG was found to be well-eluted. The eluted crude [^18^F]FSPG solution was further purified by an Alumina N cartridge and SPE column containing ENVI-Carb graphitized carbon to remove remaining [^18^F]fluoride and the impurities from the side reactions, respectively. Compared to the previous reports that require 83 min to produce [^18^F]FSPG in the TRACERlab™ and 60 min in the FASTlab™ (Shih et al. 2020; Edwards et al. 2021), our production time was greatly reduced to within 45 min in both modules. More importantly, all commercially available cartridges and resins can be used directly without any modifications in our production process, presenting it ideal protocol for cGMP manufacturing of [^18^F]FSPG for injection.

## Conclusions

We have developed highly-reliable and simplified automated methods for the routine production of [^18^F]FSPG with high activity concentration and radiochemical purity using two commercially available synthesizers. While the clinical trials using [^18^F]FSPG PET are currently underway, the automated approaches reported herein will accelerate the clinical adoption of this radiotracer and enable centralized and large-scale production of [^18^F]FSPG.

## Data Availability

All data generated or analyzed during this study are included in this manuscript.
